# 3-(4-Chloro­phen­yl)-5-(thio­phen-2-yl)-4,5-dihydro-1*H*-pyrazole-1-carbothio­amide

**DOI:** 10.1107/S1600536811054754

**Published:** 2012-01-07

**Authors:** Hoong-Kun Fun, Thitipone Suwunwong, Suchada Chantrapromma

**Affiliations:** a X-ray Crystallography Unit, School of Physics, Universiti Sains Malaysia, 11800 USM, Penang, Malaysia; b Crystal Materials Research Unit, Department of Chemistry, Faculty of Science, Prince of Songkla University, Hat-Yai, Songkhla 90112, Thailand

## Abstract

In the title pyrazoline derivative, C_14_H_12_ClN_3_S_2_, the thiophene ring is disordered over two orientations with a refined site-occupancy ratio of 0.832 (4):0.168 (4). The pyrazoline ring adopts an envelope conformation with the C atom linking the thiophene ring at the flap. The dihedral angles between the benzene ring and the major and minor components of the thiophene ring are 88.6 (3) and 85.6 (15)°, respectively while the dihedral angle between the disorder components of the ring is 3.1 (16)°. The mean plane of the pyrazoline ring makes dihedral angles of 11.86 (13), 80.1 (3) and 83.0 (15)°, respectively, with the benzene ring, and the major and minor components of the thiophene ring. An intra­molecular N(amide)—H⋯N(pyrazoline) hydrogen bond generates an *S*(5) ring motif. In the crystal, mol­ecules are linked by weak C—H⋯S and N(amide)—H⋯S inter­actions into a tape along [10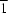]. C—H⋯π inter­actions are also observed.

## Related literature

For bond-length data, see: Allen *et al.* (1987 ▶). For hydrogen-bond motifs, see: Bernstein *et al.* (1995 ▶). For ring conformations, see: Cremer & Pople (1975 ▶). For related structures, see: Fun *et al.* (2011 ▶); Nonthason *et al.* (2011 ▶). For background to and applications of pyrazoline derivatives, see: Bai *et al.* (2007 ▶); Gong *et al.* (2011 ▶); Husain *et al.* (2008 ▶); Khode *et al.* (2009 ▶); Shoman *et al.* (2009 ▶); Taj *et al.* (2011 ▶). For the stability of the temperature controller, see: Cosier & Glazer (1986 ▶).
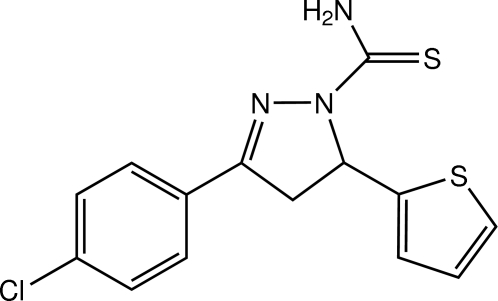


## Experimental

### 

#### Crystal data

C_14_H_12_ClN_3_S_2_
*M* *_r_* = 321.86
Monoclinic, 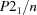

*a* = 6.7784 (3) Å
*b* = 25.2104 (11) Å
*c* = 8.4628 (4) Å
β = 90.339 (2)°
*V* = 1446.15 (11) Å^3^
*Z* = 4
Mo *K*α radiation
μ = 0.55 mm^−1^
*T* = 100 K
0.56 × 0.09 × 0.08 mm

#### Data collection

Bruker APEX DUO CCD area-detector diffractometer
Absorption correction: multi-scan (*SADABS*; Bruker, 2009 ▶) *T*_min_ = 0.749, *T*_max_ = 0.958
32828 measured reflections
4206 independent reflections
3801 reflections with *I* > 2σ(*I*)
*R*_int_ = 0.047

#### Refinement

*R*[*F* ^2^ > 2σ(*F* ^2^)] = 0.049
*wR*(*F* ^2^) = 0.138
*S* = 1.10
4206 reflections
211 parameters
10 restraints
H atoms treated by a mixture of independent and constrained refinement
Δρ_max_ = 0.33 e Å^−3^
Δρ_min_ = −0.59 e Å^−3^

### 

Data collection: *APEX2* (Bruker, 2009 ▶); cell refinement: *SAINT* (Bruker, 2009 ▶); data reduction: *SAINT*; program(s) used to solve structure: *SHELXTL* (Sheldrick, 2008 ▶); program(s) used to refine structure: *SHELXTL*; molecular graphics: *SHELXTL*; software used to prepare material for publication: *SHELXTL* and *PLATON* (Spek, 2009 ▶).

## Supplementary Material

Crystal structure: contains datablock(s) global, I. DOI: 10.1107/S1600536811054754/is5024sup1.cif

Structure factors: contains datablock(s) I. DOI: 10.1107/S1600536811054754/is5024Isup2.hkl

Supplementary material file. DOI: 10.1107/S1600536811054754/is5024Isup3.cml

Additional supplementary materials:  crystallographic information; 3D view; checkCIF report

## Figures and Tables

**Table 1:** Hydrogen-bond geometry (Å, °) *Cg*1 and *Cg*2 are the centroids of the S1*A*/C1*A*–C3*A*/C4 and S1*B*/C1*B*–C3*B*/C4 rings, respectively.

*D*—H⋯*A*	*D*—H	H⋯*A*	*D*⋯*A*	*D*—H⋯*A*
N3—H1*N*3⋯N2	0.90 (4)	2.28 (4)	2.656 (3)	105 (3)
N3—H2*N*3⋯S2^i^	0.89 (4)	2.52 (4)	3.400 (3)	170 (3)
C5—H5*A*⋯S1*A*^ii^	1.00	2.86	3.664 (3)	138
C9—H9*A*⋯*Cg*1^iii^	0.95	2.79	3.628 (4)	148
C9—H9*A*⋯*Cg*2^iii^	0.95	2.77	3.595 (18)	145

Symmetry codes: (i) 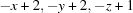; (ii) 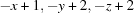; (iii) 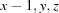.

**Fig. 1.**
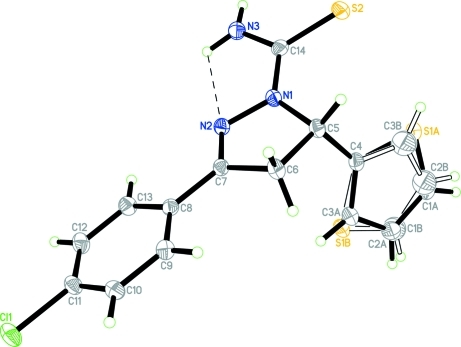
The molecular structure of the title compound, showing 45% probability displacement ellipsoids and the atom-numbering scheme. Open bond show the minor B component. Intramolecular N—H···N hydrogen bond was shown as dash line.

**Fig. 2.**
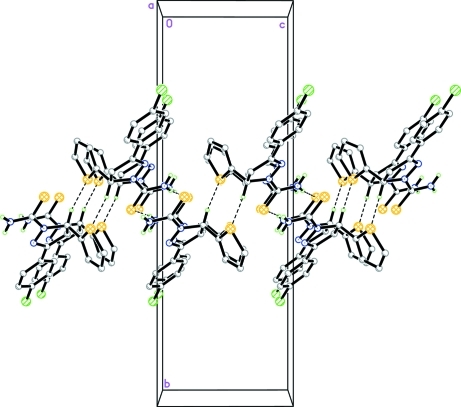
The crystal packing of the title compound viewed along the a axis. Only the major component was shown. For clarify, only H atoms involved in hydrogen bonds were shown. Hydrogen bonds were shown as dashed lines.

## supplementary crystallographic information

### Comment

The synthesis of pyrazoline derivatives which contain 5-membered heterocyclic structure have attracted a lot of interests in many fields, for example as in medicinal chemistry owing to their biological properties such as antiamoebic (Husain *et al.*, 2008), anti-inflammatory (Shoman *et al.*, 2009), analgesic (Khode *et al.*, 2009) and antioxidant (Taj *et al.*, 2011) activities, as well as in fluorescence (Bai *et al.*, 2007; Gong *et al.*, 2011) studies. Our on-going research on biological activities and fluorescent property of pyrazoline derivatives has led us to synthesize the title compound (I) in order to compare its biological activity with the related compounds (Fun *et al.*, 2011; Nonthason *et al.*, 2011).

In the molecule of (I), C_14_H_12_ClN_3_S_2_, the thiophene ring is disordered over two positions with the refined site-occupancy ratio of 0.832 (4):0.168 (4). The dihedral angles between the benzene and the major and minor components of the thiophene rings are 88.6 (3) and 85.6 (15)° respectively. The pyrazoline ring is in an envelope conformation [pucker atom at C5 with deviation of -0.125 (3) Å] with puckering parameter Q = 0.206 (3) Å and φ = 137.6 (7)° (Cremer & Pople, 1975). The dihedral angle between the mean plane through pyrazoline ring and the benzene ring is 11.86 (13)°, whereas these values are 80.1 (3) and 83.0 (15)° between the pyrazoline and the major and minor components of the thiophene ring. The carbothioamide unit lies almost on the same plane with pyrazoline ring as can be indicated by the torsion angles N2–N1–C14–N3 = -3.3 (4)° and C5–N1–C14–S2 = 0.4 (3)°. Intramolecular N3—H1N3···N2 hydrogen bond generate an S(5) ring motif (Bernstein *et al.*, 1995). Bond distances of (I) are in normal range (Allen *et al.*, 1987)

In the crystal packing, (Fig. 2), the molecules are linked by weak C5—H5A···S1A intermolecular interactions (Table 1) into cyclic centrosymmetric *R*^2^_2_(8) dimers (Bernstein *et al.*, 1995). These dimers are further linked by N3—H2N3···S2 hydrogen bonds (Table 1) into a tape along the [101] direction (Fig. 2). The crystal is stabilized by N—H···S hydrogen bonds together with weak C—H···S and C—H···π interactions (Table 1).

### Experimental

The title compound was synthesized by dissolving (*E*)-1-(4-chlorophenyl)-3-(2-thienyl)prop-2-en-1-one (0.25 g, 1.0 mmol) in a solution of KOH (0.06 g, 1.0 mmol) in ethanol (20 ml). An excess thiosemicarbazide (0.14 g, 1.5 mmol) in ethanol (20 ml) was then added, and the reaction mixture was vigorously stirred and refluxed for 4 h. The pale-yellow solid of the title compound obtained after cooling of the reaction was filtered off under vacuum. Pale yellow needle-shaped single crystals of the title compound suitable for *X*-ray structure determination were recrystalized from CH_3_OH/CH_2_Cl_2_ (1:1 *v*/*v*) by slow evaporation of the solvent at room temperature after several days.

### Refinement

Amide H atoms were located in a difference map and refined isotropically. The remaining H atoms were positioned geometrically and allowed to ride on their parent atoms, with d(C—H) = 0.95 Å for aromatic and 0.99 Å for CH_2_ atoms. The *U*_iso_ values were constrained to be 1.2*U*_eq_ of the carrier atoms. The thiophene ring is disordered over two positions with the refined site-occupancy ratio of 0.832 (4):0.168 (4). In the final refinement, distances restraint was used. The highest residual electron density peak is located at 1.35 Å from Cl1 and the deepest hole is located at 0.52 Å from Cl1. The crystal was a pseudo-merohedral twin and the structure was refined with the twin law (-1 0 0 0 -1 0 0 0 1). The BASF was refined to 0.138 (1).

### Crystal data

**Table:**

C_14_H_12_ClN_3_S_2_	*F*(000) = 664
*M**_r_* = 321.86	*D*_x_ = 1.478 Mg m^−^^3^
Monoclinic, *P*2_1_/*n*	Mo *K*α radiation, λ = 0.71073 Å
Hall symbol: -P 2yn	Cell parameters from 4206 reflections
*a* = 6.7784 (3) Å	θ = 0.8–30.0°
*b* = 25.2104 (11) Å	µ = 0.55 mm^−^^1^
*c* = 8.4628 (4) Å	*T* = 100 K
β = 90.339 (2)°	Needle, pale-yellow
*V* = 1446.15 (11) Å^3^	0.56 × 0.09 × 0.08 mm
*Z* = 4	

### Data collection

**Table:**

Bruker APEX DUO CCD area-detector diffractometer	4206 independent reflections
Radiation source: sealed tube	3801 reflections with *I* > 2σ(*I*)
graphite	*R*_int_ = 0.047
φ and ω scans	θ_max_ = 30.0°, θ_min_ = 0.8°
Absorption correction: multi-scan (*SADABS*; Bruker, 2009)	*h* = −9→9
*T*_min_ = 0.749, *T*_max_ = 0.958	*k* = −35→35
32828 measured reflections	*l* = −11→11

### Refinement

**Table:**

Refinement on *F*^2^	Primary atom site location: structure-invariant direct methods
Least-squares matrix: full	Secondary atom site location: difference Fourier map
*R*[*F*^2^ > 2σ(*F*^2^)] = 0.049	Hydrogen site location: inferred from neighbouring sites
*wR*(*F*^2^) = 0.138	H atoms treated by a mixture of independent and constrained refinement
*S* = 1.10	*w* = 1/[σ^2^(*F*_o_^2^) + (0.0644*P*)^2^ + 1.9543*P*] where *P* = (*F*_o_^2^ + 2*F*_c_^2^)/3
4206 reflections	(Δ/σ)_max_ = 0.002
211 parameters	Δρ_max_ = 0.33 e Å^−^^3^
10 restraints	Δρ_min_ = −0.59 e Å^−^^3^

### Special details

**Table:**

Experimental. The crystal was placed in the cold stream of an Oxford Cryosystems Cobra open-flow nitrogen cryostat (Cosier & Glazer, 1986) operating at 100.0 (1) K.
Geometry. All esds (except the esd in the dihedral angle between two l.s. planes) are estimated using the full covariance matrix. The cell esds are taken into account individually in the estimation of esds in distances, angles and torsion angles; correlations between esds in cell parameters are only used when they are defined by crystal symmetry. An approximate (isotropic) treatment of cell esds is used for estimating esds involving l.s. planes.
Refinement. Refinement of F^2^ against ALL reflections. The weighted R-factor wR and goodness of fit S are based on F^2^, conventional R-factors R are based on F, with F set to zero for negative F^2^. The threshold expression of F^2^ > 2sigma(F^2^) is used only for calculating R-factors(gt) etc. and is not relevant to the choice of reflections for refinement. R-factors based on F^2^ are statistically about twice as large as those based on F, and R- factors based on ALL data will be even larger.

### Fractional atomic coordinates and isotropic or equivalent isotropic displacement parameters (Å^2^)

**Table:**

	*x*	*y*	*z*	*U*_iso_*/*U*_eq_	Occ. (<1)
Cl1	−0.33129 (11)	0.74126 (3)	0.43185 (9)	0.02922 (17)	
S2	0.81787 (10)	1.01564 (3)	0.70895 (8)	0.02383 (16)	
N1	0.5530 (3)	0.93930 (8)	0.6751 (3)	0.0187 (4)	
N2	0.4447 (3)	0.90237 (8)	0.5877 (2)	0.0182 (4)	
C9	−0.0433 (4)	0.85079 (10)	0.6843 (3)	0.0200 (5)	
H9A	−0.0680	0.8721	0.7747	0.024*	
N3	0.7384 (4)	0.95592 (10)	0.4561 (3)	0.0264 (5)	
C4	0.5995 (4)	0.91382 (10)	0.9528 (3)	0.0190 (4)	
S1A	0.81128 (18)	0.93898 (4)	1.03729 (11)	0.0196 (2)	0.832 (4)
C1A	0.8584 (10)	0.8815 (2)	1.1384 (9)	0.0242 (10)	0.832 (4)
H1AA	0.9681	0.8764	1.2071	0.029*	0.832 (4)
C2A	0.7184 (16)	0.8437 (3)	1.1078 (12)	0.0289 (15)	0.832 (4)
H2AA	0.7203	0.8090	1.1519	0.035*	0.832 (4)
C3A	0.5701 (18)	0.8623 (3)	1.0024 (14)	0.0263 (16)	0.832 (4)
H3AA	0.4610	0.8413	0.9692	0.032*	0.832 (4)
S1B	0.544 (2)	0.8499 (4)	0.9965 (18)	0.0243 (19)	0.168 (4)
C1B	0.751 (7)	0.8414 (15)	1.110 (8)	0.038 (14)*	0.168 (4)
H1BA	0.7880	0.8089	1.1588	0.046*	0.168 (4)
C2B	0.856 (7)	0.8873 (15)	1.122 (7)	0.041 (9)*	0.168 (4)
H2BA	0.9726	0.8912	1.1832	0.049*	0.168 (4)
C3B	0.770 (4)	0.9289 (10)	1.031 (4)	0.041 (9)*	0.168 (4)
H3BA	0.8240	0.9636	1.0250	0.049*	0.168 (4)
C5	0.4780 (4)	0.94575 (10)	0.8380 (3)	0.0186 (4)	
H5A	0.4751	0.9840	0.8691	0.022*	
C6	0.2675 (4)	0.92375 (11)	0.8167 (3)	0.0210 (5)	
H6A	0.2293	0.9009	0.9067	0.025*	
H6B	0.1698	0.9527	0.8047	0.025*	
C7	0.2870 (3)	0.89198 (9)	0.6660 (3)	0.0170 (4)	
C8	0.1379 (4)	0.85466 (9)	0.6075 (3)	0.0174 (4)	
C10	−0.1881 (4)	0.81612 (10)	0.6300 (3)	0.0207 (5)	
H10A	−0.3117	0.8140	0.6819	0.025*	
C11	−0.1501 (4)	0.78485 (10)	0.4997 (3)	0.0213 (5)	
C12	0.0296 (4)	0.78746 (11)	0.4212 (3)	0.0238 (5)	
H12A	0.0537	0.7655	0.3321	0.029*	
C13	0.1730 (4)	0.82256 (10)	0.4750 (3)	0.0219 (5)	
H13A	0.2957	0.8249	0.4217	0.026*	
C14	0.6989 (4)	0.96747 (10)	0.6069 (3)	0.0205 (5)	
H1N3	0.673 (6)	0.9294 (15)	0.408 (5)	0.030 (9)*	
H2N3	0.851 (6)	0.9675 (15)	0.416 (4)	0.032 (9)*	

### Atomic displacement parameters (Å^2^)

**Table:**

	*U*^11^	*U*^22^	*U*^33^	*U*^12^	*U*^13^	*U*^23^
Cl1	0.0269 (3)	0.0216 (3)	0.0391 (4)	−0.0052 (2)	−0.0061 (3)	−0.0048 (3)
S2	0.0228 (3)	0.0227 (3)	0.0259 (3)	−0.0056 (2)	−0.0034 (2)	0.0011 (2)
N1	0.0178 (9)	0.0199 (10)	0.0183 (9)	−0.0026 (7)	−0.0012 (7)	−0.0010 (8)
N2	0.0172 (9)	0.0188 (9)	0.0186 (9)	−0.0006 (7)	−0.0026 (7)	0.0008 (7)
C9	0.0199 (11)	0.0196 (11)	0.0204 (11)	0.0018 (9)	−0.0006 (8)	−0.0018 (9)
N3	0.0265 (11)	0.0329 (13)	0.0198 (10)	−0.0107 (10)	0.0001 (9)	0.0018 (9)
C4	0.0185 (10)	0.0215 (11)	0.0171 (10)	−0.0012 (8)	0.0002 (8)	−0.0041 (9)
S1A	0.0192 (4)	0.0206 (4)	0.0188 (4)	0.0009 (4)	−0.0021 (3)	−0.0030 (3)
C1A	0.029 (2)	0.024 (2)	0.019 (2)	0.0046 (15)	−0.0052 (13)	−0.0011 (15)
C2A	0.037 (3)	0.023 (2)	0.027 (3)	−0.003 (2)	−0.006 (2)	0.0051 (14)
C3A	0.028 (3)	0.027 (4)	0.024 (2)	−0.009 (3)	−0.005 (2)	0.000 (3)
S1B	0.026 (4)	0.022 (4)	0.025 (3)	−0.007 (3)	−0.007 (2)	0.000 (3)
C5	0.0171 (10)	0.0196 (11)	0.0191 (10)	−0.0004 (8)	0.0004 (8)	−0.0037 (8)
C6	0.0185 (11)	0.0222 (11)	0.0224 (11)	−0.0010 (9)	0.0003 (9)	−0.0052 (9)
C7	0.0172 (10)	0.0163 (10)	0.0174 (10)	0.0007 (8)	−0.0031 (8)	0.0001 (8)
C8	0.0196 (11)	0.0150 (10)	0.0175 (10)	0.0009 (8)	−0.0020 (8)	0.0010 (8)
C10	0.0182 (10)	0.0182 (11)	0.0257 (12)	−0.0002 (9)	−0.0008 (9)	0.0008 (9)
C11	0.0218 (11)	0.0158 (10)	0.0263 (12)	−0.0019 (9)	−0.0047 (9)	−0.0003 (9)
C12	0.0270 (12)	0.0211 (12)	0.0231 (12)	−0.0001 (10)	−0.0027 (10)	−0.0062 (9)
C13	0.0219 (11)	0.0216 (11)	0.0222 (11)	0.0004 (10)	0.0011 (9)	−0.0028 (9)
C14	0.0200 (11)	0.0213 (11)	0.0201 (11)	−0.0015 (9)	−0.0029 (9)	0.0045 (9)

### Geometric parameters (Å, °)

**Table:**

Cl1—C11	1.743 (3)	C2A—H2AA	0.9500
S2—C14	1.692 (3)	C3A—H3AA	0.9500
N1—C14	1.350 (3)	S1B—C1B	1.71 (2)
N1—N2	1.395 (3)	C1B—C2B	1.363 (18)
N1—C5	1.481 (3)	C1B—H1BA	0.9500
N2—C7	1.288 (3)	C2B—C3B	1.42 (2)
C9—C10	1.390 (3)	C2B—H2BA	0.9500
C9—C8	1.397 (3)	C3B—H3BA	0.9500
C9—H9A	0.9500	C5—C6	1.541 (3)
N3—C14	1.338 (3)	C5—H5A	1.0000
N3—H1N3	0.90 (4)	C6—C7	1.512 (3)
N3—H2N3	0.89 (4)	C6—H6A	0.9900
C4—C3A	1.379 (8)	C6—H6B	0.9900
C4—C3B	1.381 (18)	C7—C8	1.465 (3)
C4—C5	1.503 (3)	C8—C13	1.405 (3)
C4—S1B	1.696 (10)	C10—C11	1.381 (4)
C4—S1A	1.721 (3)	C10—H10A	0.9500
S1A—C1A	1.713 (6)	C11—C12	1.392 (4)
C1A—C2A	1.368 (6)	C12—C13	1.389 (4)
C1A—H1AA	0.9500	C12—H12A	0.9500
C2A—C3A	1.421 (12)	C13—H13A	0.9500
			
C14—N1—N2	120.6 (2)	C4—C3B—H3BA	123.4
C14—N1—C5	126.6 (2)	C2B—C3B—H3BA	123.4
N2—N1—C5	112.60 (19)	N1—C5—C4	110.7 (2)
C7—N2—N1	107.4 (2)	N1—C5—C6	100.05 (19)
C10—C9—C8	120.7 (2)	C4—C5—C6	112.7 (2)
C10—C9—H9A	119.6	N1—C5—H5A	111.0
C8—C9—H9A	119.6	C4—C5—H5A	111.0
C14—N3—H1N3	120 (3)	C6—C5—H5A	111.0
C14—N3—H2N3	118 (3)	C7—C6—C5	101.7 (2)
H1N3—N3—H2N3	119 (4)	C7—C6—H6A	111.4
C3A—C4—C3B	103.6 (13)	C5—C6—H6A	111.4
C3A—C4—C5	128.4 (5)	C7—C6—H6B	111.4
C3B—C4—C5	128.0 (11)	C5—C6—H6B	111.4
C3B—C4—S1B	110.0 (11)	H6A—C6—H6B	109.3
C5—C4—S1B	121.9 (5)	N2—C7—C8	122.0 (2)
C3A—C4—S1A	110.0 (5)	N2—C7—C6	113.8 (2)
C5—C4—S1A	121.56 (18)	C8—C7—C6	124.2 (2)
S1B—C4—S1A	116.4 (5)	C9—C8—C13	119.0 (2)
C1A—S1A—C4	92.7 (2)	C9—C8—C7	119.6 (2)
C2A—C1A—S1A	111.6 (4)	C13—C8—C7	121.4 (2)
C2A—C1A—H1AA	124.2	C11—C10—C9	119.2 (2)
S1A—C1A—H1AA	124.2	C11—C10—H10A	120.4
C1A—C2A—C3A	112.1 (5)	C9—C10—H10A	120.4
C1A—C2A—H2AA	124.0	C10—C11—C12	121.4 (2)
C3A—C2A—H2AA	124.0	C10—C11—Cl1	119.3 (2)
C4—C3A—C2A	113.5 (6)	C12—C11—Cl1	119.3 (2)
C4—C3A—H3AA	123.2	C13—C12—C11	119.1 (2)
C2A—C3A—H3AA	123.2	C13—C12—H12A	120.4
C4—S1B—C1B	93.5 (11)	C11—C12—H12A	120.4
C2B—C1B—S1B	111.0 (19)	C12—C13—C8	120.5 (2)
C2B—C1B—H1BA	124.5	C12—C13—H13A	119.8
S1B—C1B—H1BA	124.5	C8—C13—H13A	119.8
C1B—C2B—C3B	112 (2)	N3—C14—N1	116.4 (2)
C1B—C2B—H2BA	124.0	N3—C14—S2	123.1 (2)
C3B—C2B—H2BA	124.0	N1—C14—S2	120.50 (19)
C4—C3B—C2B	113.3 (17)		
			
C14—N1—N2—C7	−163.4 (2)	S1B—C4—C5—N1	−89.2 (7)
C5—N1—N2—C7	12.0 (3)	S1A—C4—C5—N1	86.6 (2)
C3A—C4—S1A—C1A	−0.4 (7)	C3A—C4—C5—C6	20.2 (8)
C3B—C4—S1A—C1A	9(13)	C3B—C4—C5—C6	−161.3 (19)
C5—C4—S1A—C1A	−178.3 (4)	S1B—C4—C5—C6	21.9 (7)
S1B—C4—S1A—C1A	−2.2 (7)	S1A—C4—C5—C6	−162.34 (18)
C4—S1A—C1A—C2A	0.7 (8)	N1—C5—C6—C7	19.1 (2)
S1A—C1A—C2A—C3A	−0.8 (14)	C4—C5—C6—C7	−98.4 (2)
C3B—C4—C3A—C2A	−1.1 (19)	N1—N2—C7—C8	179.6 (2)
C5—C4—C3A—C2A	177.7 (7)	N1—N2—C7—C6	2.6 (3)
S1B—C4—C3A—C2A	165 (11)	C5—C6—C7—N2	−14.8 (3)
S1A—C4—C3A—C2A	0.0 (12)	C5—C6—C7—C8	168.2 (2)
C1A—C2A—C3A—C4	0.5 (16)	C10—C9—C8—C13	−0.6 (4)
C3A—C4—S1B—C1B	−17 (10)	C10—C9—C8—C7	179.3 (2)
C3B—C4—S1B—C1B	−3(3)	N2—C7—C8—C9	−170.3 (2)
C5—C4—S1B—C1B	174 (3)	C6—C7—C8—C9	6.4 (4)
S1A—C4—S1B—C1B	−2(3)	N2—C7—C8—C13	9.6 (4)
C4—S1B—C1B—C2B	3(6)	C6—C7—C8—C13	−173.7 (2)
S1B—C1B—C2B—C3B	−3(8)	C8—C9—C10—C11	0.8 (4)
C3A—C4—C3B—C2B	4(4)	C9—C10—C11—C12	−0.3 (4)
C5—C4—C3B—C2B	−175 (3)	C9—C10—C11—Cl1	179.84 (19)
S1B—C4—C3B—C2B	2(4)	C10—C11—C12—C13	−0.4 (4)
S1A—C4—C3B—C2B	−168 (16)	Cl1—C11—C12—C13	179.5 (2)
C1B—C2B—C3B—C4	0(7)	C11—C12—C13—C8	0.5 (4)
C14—N1—C5—C4	−86.0 (3)	C9—C8—C13—C12	−0.1 (4)
N2—N1—C5—C4	98.9 (2)	C7—C8—C13—C12	−179.9 (2)
C14—N1—C5—C6	154.9 (2)	N2—N1—C14—N3	−3.3 (4)
N2—N1—C5—C6	−20.1 (3)	C5—N1—C14—N3	−178.0 (2)
C3A—C4—C5—N1	−90.9 (8)	N2—N1—C14—S2	175.10 (17)
C3B—C4—C5—N1	87.6 (19)	C5—N1—C14—S2	0.4 (3)

### Hydrogen-bond geometry (Å, °)

**Table:**

*Cg*1 and *Cg*2 are the centroids of the S1*A*/C1*A*–C3*A*/C4 and S1*B*/C1*B*–C3*B*/C4 rings, respectively.

**Table:**

*D*—H···*A*	*D*—H	H···*A*	*D*···*A*	*D*—H···*A*
N3—H1N3···N2	0.90 (4)	2.28 (4)	2.656 (3)	105 (3)
N3—H2N3···S2^i^	0.89 (4)	2.52 (4)	3.400 (3)	170 (3)
C5—H5A···S1A^ii^	1.00	2.86	3.664 (3)	138
C9—H9A···Cg1^iii^	0.95	2.79	3.628 (4)	148
C9—H9A···Cg2^iii^	0.95	2.77	3.595 (18)	145

Symmetry codes: (i) −*x*+2, −*y*+2, −*z*+1; (ii) −*x*+1, −*y*+2, −*z*+2; (iii) *x*−1, *y*, *z*.
